# Predictors of breastfeeding duration in a predominantly Māori population in New Zealand

**DOI:** 10.1186/s12887-018-1274-9

**Published:** 2018-09-12

**Authors:** Kathy M. Manhire, Sheila M. Williams, David Tipene-Leach, Sally A. Baddock, Sally Abel, Angeline Tangiora, Raymond Jones, Barry J. Taylor

**Affiliations:** 1 Department of Women’s and Children’s Health, Dunedin School of Medicine, University of Otago, Dunedin, New Zealand; 2 Faculty of Education, Humanities and Health Sciences, Eastern Institute of Technology, Hawke’s Bay, New Zealand; 3 Department of Preventive & Social Medicine, Dunedin School of Medicine, University of Otago, Dunedin, New Zealand; 4 School of Midwifery, Otago Polytechnic, Dunedin, New Zealand; 5 Kaupapa Consulting Ltd, Napier, Napier, New Zealand

**Keywords:** Infant nutrition, Lactation, Maternal knowledge, Pacifier

## Abstract

**Background:**

Although breastfeeding duration in New Zealand’s indigenous Māori is shorter than in non-Māori, we know little about barriers or motivators of breastfeeding in this community. The aim of this analysis was to identify predictors for extended duration of breastfeeding amongst participants drawn from predominantly Māori communities in regional Hawke’s Bay.

**Methods:**

Mother/baby dyads were recruited from two midwifery practices serving predominantly Māori women in mostly deprived areas, for a randomised controlled trial comparing the risks and benefits of an indigenous sleeping device (*wahakura)* and a bassinet. Questionnaires were administered at baseline (pregnancy) and at one, three and six months postnatal. Several questions relating to breastfeeding and factors associated with breastfeeding were included. The data from both groups were pooled to examine predictors of breastfeeding duration.

**Results:**

Māori comprised 70.5% of the 197 participants recruited. The median time infants were fully breastfed was eight weeks and Māori women were more likely to breastfeed for a shorter duration than New Zealand European women with an odds-ratio (OR) of 0.45 (95% CI 0.24, 0.85). The key predictors for extended duration of breastfeeding were the strong support of the mother’s partner (OR = 3.64, 95% CI 1.76, 7.55) or her mother for breastfeeding (OR = 2.47, 95% CI 1.27, 4.82), longer intended duration of maternal breastfeeding (OR = 1.02, 95% CI 1.00, 1.03) and being an older mother (OR = 1.07, 95% CI 1.02, 1.12). The key predictors for shorter duration of breastfeeding were pacifier use (OR = 0.28, 95% CI 0.17, 0.46), daily cigarette smoking (OR = 0.51, 95% CI 0.37, 0.69), alcohol use (OR = 0.54, 95% CI 0.31, 0.93) and living in a more deprived area (OR 0.40, 95% CI 0.22, 0.72).

**Conclusions:**

Breastfeeding duration in this group of mainly Māori women was shorter than the national average. Increasing the duration of breastfeeding by these mothers could be further facilitated by ante and postnatal education involving their own mothers and their partners in the support of breastfeeding and by addressing pacifier use, smoking and alcohol use.

## Background

Breastfeeding, including the consumption of expressed breast milk, is the normative standard for infant feeding and nutrition [1] and is significant in the prevention of disease in later life [2, 3] particularly where breastfeeding is exclusive and lasts for at least six months [4]. In New Zealand, Māori women generally live in more deprived and low income circumstances and have a shorter duration of breastfeeding than non-Māori and reducing this disparity among the indigenous population is a major focus for the New Zealand Ministry of Health [5]. Therefore, understanding the barriers and motivators to extended breastfeeding among Māori is important.

A range of demographic factors are known to affect breastfeeding duration. These include maternal age [6], socioeconomic status [7], ethnicity [8] and cultural group or indigeneity [9]. Factors known to extend breastfeeding include maternal breastfeeding knowledge and beliefs [10, 11], maternal intention to breastfeed [12] and family support for breastfeeding behaviour [7]. On the other hand, those associated with a shorter duration of breastfeeding include factors, such as maternal smoking [13, 14], maternal alcohol use [15] and maternal depression [16, 17], and the use of a pacifier [18, 19]. The effect of the early introduction of baby to solids is unclear, needing both a definition of ‘early’ and being clearly differentiated from introduction of formula [20, 21].

A number of qualitative studies in New Zealand have examined motivators for and barriers to extended breastfeeding duration among Māori women. One study found factors that extended breastfeeding among young Māori mothers included maternal perception of breastfeeding as natural and easy, early stage breastfeeding support, being determined to breastfeed despite adversity and informed decision-making around the use of infant formula [22]. Negative influences on breastfeeding from two larger qualitative studies with Māori mothers and *whānau* (extended family) included: difficulty establishing early breastfeeding, insufficient professional support, the perception of inadequate milk supply and the need to return to work [23]; the lack of culturally pertinent breastfeeding information, confusion about the impacts on the baby when smoking during breastfeeding, uncertainty about the safety of bedsharing and a perceived lack of acceptability of breastfeeding in public [24]. Some commentators have argued that the breakdown in whānau breastfeeding norms and low Māori breastfeeding rates are attributable to the impacts of colonization, including the influence of mid twentieth century infant welfare programmes encouraging the use of infant formula [25].

This paper adds to these qualitative findings by reporting on results from a recent quantitative study which enables us to identify and quantify predictors for extended breastfeeding in a sample of predominantly Māori women from more deprived communities in New Zealand.

## Methods

Healthy mother/baby dyads from singleton pregnancies were recruited between June 2011 and April 2013 from two midwifery practices supporting predominantly Māori families in areas of Hawke’s Bay, most of which had a high New Zealand deprivation index score [26]. At the prenatal visit they were entered into a two arm, randomised controlled trial comparing the risks and benefits of an indigenous infant sleep device, the *wahakura* (a 36 × 72 cm flax bassinet), and a stand-alone bassinet. A baseline survey was completed in pregnancy with further surveys at one, three and six months postnatally. The complete methodology, including recruitment strategy and sample size for breastfeeding, has been published previously [27].

Data were collected by a local Māori researcher using machine readable questionnaires (HP TeleForm ©2014 Hewlett-Packard Development Company, L.P.). The one and three month questionnaires were completed as face-to-face interviews with the babies’ mothers and the six-month questionnaire was administered by telephone. Participants were given a NZ $25 voucher on completion of each of the three and six month interviews.

Self-identified ethnicity and a range of other demographic data were collected at baseline as were questions about the mother’s intention to breastfeed (‘do you plan to breastfeed’ and ‘to what age do you plan to exclusively breastfeed’) and her knowledge of breastfeeding (‘recommended age for exclusive breastfeeding’ and ‘recommended age for introduction of solids’). The survey also included a question about whether the mother herself was breastfed as a baby.

In the three postnatal surveys, questions relating to breastfeeding behaviour, including the use of expressed breast milk, were asked; (‘currently breastfeeding your baby for any time’ and ‘currently fully breastfeeding your baby’). Supplementary questions enquired about the age at which babies ‘stopped breastfeeding’, ‘stopped fully breastfeeding’, ‘had any milk or food that was not breast milk’ and when they ‘began solids’. This information allowed the breastfeeding practice to later be reclassified as: exclusive (breastmilk and prescribed medicines from birth); full (breastmilk and minimal water/water-based drinks or prescribed medicines in the last 48 h); partial (breastmilk and solid or semi-solid food which may include infant formula or non- human milk); and no breastmilk at all [28]. There were open questions about the type of food and solids given to babies. Questions relating to sleep location (shared parents’ bedroom and shared mother’s bed), maternal sleep quality and quantity (on a 4 point scale) [29] and a question about pacifier (dummy) use, (‘Did baby use dummy in the past week?’) were also asked at these times.

At both the baseline and one month surveys, participants were asked about how supportive (‘strongly supportive, supportive, neutral, not supportive, not at all supportive’) their partner, and also their mother would be or was of them breastfeeding their baby. At one month questions were also asked about tobacco use (tobacco smoking status and daily use) and alcohol consumption (‘on the days when you drink alcohol, how many drinks do you usually have?’) As we have no information about the frequency of alcohol consumption we limited the analysis to alcohol use at one month. Maternal depression (Edinburgh Postnatal Depression Scale score > 10) was asked about using the Edinburgh Postnatal Depression Scale (EPDS) [30] at baseline, and then at three and six months.

### Statistical analyses

The proportional-odds cumulative logit model was used to analyze most of the data [31]. This provides an odds-ratio or, in this case, a proportional-odds (OR) which compares, in a cumulative way, people who are in groups greater than *k* versus those who are in groups less than or equal to *k*, where *k* is the level of the response variable. This can be interpreted as for a one unit change in the predictor variable, the odds for cases in a group that is greater than *k* versus less than or equal to *k* are the proportional-odds times larger. The data was analyzed using a Stata (Stata Statistical Software: Release 13) procedure, which provides a check of the underlying assumptions for proportional-odds models. As some variables, for instance the use of a pacifier, were collected in the same way at all three postnatal interviews, we used a logistic model which accounted for the time varying covariates [31].

Ethics approval to conduct this study was granted by the New Zealand Central Region Ethics Committee (CEN/10.12.054). All participants were provided with a written Participant Information sheet and a Consent Form to be signed.

## Results

One hundred and ninety-seven mother/baby dyads were recruited out of 600 eligible mothers giving a recruitment rate of 33%. Seventy percent self-identified as Māori and two-thirds were from quintile five areas, those with highest deprivation index score [27]. The retention rate at six months was 88%.

The frequency of full, partial and no breastfeeding and the age of the infant at the time of the one, three and six month surveys, alongside information about the frequency of feeding ‘foods not breast milk’ and the ‘introduction of solids’ can be seen in Table 1. Notably, by one month over half (53.3%) were having ‘food not breast milk’ and some (4.9%) were having ‘solids introduced’. The open questions revealed that ‘food other than breast milk’ was mainly some form of infant formula and the ‘solids introduced’ included Weetbix, Farex, baby rice or yoghurt. By three months, those foods included canned baby food, mashed vegetables, pureed fruit and custard and, in the case of one baby, strawberries dipped in chocolate. Although foods such as avocado, biscuits, and chewed sausage were given to one or two babies before six months, the majority of babies were given baby cereal, canned food or mashed fruit or vegetables.

**Table 1:** Mean assessment age, frequency of breastfeeding/other foods and estimated breastfeeding prevalence at 4, 13 and 26 weeks

Assessment	1 month *N* = 182	3 month *N* = 183	6 month *n* = 173
Age at assessment (days) Mean (sd)	51 (25)	108 (29)	203 (33)
Breastfeeding	n (%)	n (%)	n (%)
Full	92 (50.5)	66 (36.0)	28 (16.2)
Partial	46 (25.3)	49 (26.8)	52 (30.1)
None	44 (24.2)	72 (39.3)	93 (53.7)
Food not breast milk introduced	97 (53.3)	114 (62.3)	154 (89.3)
Solids introduced	9 (4.9)	42 (22.9)	166 (93.4)
Reclassification of breastfeeding ^a^	4 weeks	13 weeks	26 weeks
Exclusive	75 (40.1)	35 (18.7)	0
Full	31 (16.6)	28 (15.0)	29 (15.5)
Partial	33 (17.7)	44 (23.5)	53 (28.3)
None	48 (25.7)	80 (42.8)	105 (56.1)

^a^Based on information gained at the 1, 3 and 6 month interviews

Because the mothers were asked about the timing of stopping fully or partially breastfeeding their infants as well as the timing of the introduction of foods other than breast milk, we were able to estimate that the median time the infants were breast fed was eight weeks (IQR 3–20). As the age at assessment did not match the nominated times of one, three and six months we estimated the frequency of exclusive, fully and partial breastfeeding at four, 13 and 26 weeks. Table 1 shows that three-quarters of the sample were having some breastmilk at both the one month assessment (75.8%) and at the reclassified four weeks (74.6%). Of these, 40.1% were exclusively breastfeeding and this fell to 20% by 13 weeks and to zero by 26 weeks. Full breastfeeding fell from 56.6 to 15.5% over that time period.

For further analysis the sample was divided into four groups related to the length of time they were fully breastfed: babies who were breastfed for less than four weeks (*n* = 95), babies breastfed for four weeks or more but less than 13 weeks (*n* = 31), babies breastfed for 13 weeks or more but less than 26 weeks (*n* = 33) and babies still fully breastfeeding at the age of 26 weeks (*n* = 28). We used the proportional-odds ratio (OR) to estimate the association between these four breastfeeding groups and a number of important demographic and social characteristics of the mothers and babies. These are shown in Table 2. The proportional-odds for maternal age indicates that older mothers were more likely to breastfeed their babies for longer (OR = 1.07; 95% CI 1.02, 1.12); that mothers living in a more deprived neighbourhood were likely to breast-feed their baby for a shorter period than those living in a more advantaged neighbourhood (OR = 0.40; 95% CI 0.22, 0.72) and that non-Māori women breastfed their babies for longer than Māori (OR = 2.22; 95% CI 1.17, 4.21). Babies heavier at birth were also more likely to be breastfed for longer the odds-ratio for a 100 g difference in birthweight was OR = 1.07 (95% CI 1.01,1.13). Mothers level of education, having a partner, number of children, gender of child, mode of birth, knowledge of *iwi* (tribal) origin, and being randomized to receive a wahakura were not related to breastfeeding duration.

**Table 2:** Means and frequency for demographic and birth variables for four ‘fully breastfeeding’ groups

	Full breastfeeding stopped before 4 weeks *N* = 95	Full breastfeeding stopped at/after 4 weeks but before 13 weeks *N* = 31	Full breastfeeding stopped at/after 13 weeks but before 26 weeks *N* = 33	Full breastfeeding stopped at/after 26 weeks *N* = 28	OR (95% CI)	*p*
Mothers mean age	25.1 (6.05)	26.1 (6.09)	27.8 (6.43)	28.8 (6.55)	1.07 (1.02, 1.12)	0.002
(sd) Mothers education	n (%)	n (%)	n (%)	n (%)		
Completed year 11	48 (56)	16 (19)	13 (15)	9 (10)		
Completed year 13	20 (51)	5 (13)	8 (21)	6 (15)		
Some tertiary qual	27 (44)	10 (16)	12 (19)	13 (21)	1.34 (0.99, 1.82)^b^	0.060
Has partner						
No	9(9)	2 (6)	2(6)	2 (7)		
Yes	86 (91)	29 (94)	31 (94)	26 (93)	1.43 (0.50, 4.04)	0.758
Number of children						
0	23 (24)	12 (39)	9 (27)	3 (11)		
1	25 (26)	7 (23)	15 (45)	11 (39)		
2	22 (23)	5 (16)	7 (21)	8 (29)		
3 or 4	25 (26)	7 (23)	2 (6)	6 (21)	0.91 (0.74,1.14)^b^	0.415
NZDEP						
1–8	19(22)	5(16)	14(62)	14(50)		
9,10	74 (78)	26 (84)	19 (58)	14 (50)	0.40 (0.22, 0.72)	0.003
Maternal ethnicity						
Maori	73 (77)	23 (74)	18 (55)	17 (61)	1.00	
European/other	15 (16)	7 (23)	11 (33)	9 (32)	2.22 (1.17, 4.21)	0.014
Pacific Island	7 (7)	1 (3)	4 (12)	2 (7)	1.43 (0.51, 4.04)	0.501
Knows iwi (tribe)						
No	67(91)	20(92)	17 (11)	14 (18)		
Yes	7 (9)	24 (8)	2 (89)	3/ (82)	0.68 (0.24, 1.97)	0.484
Sex of infant						
Boy	46(48)	28(58)	16(48)	14(50)		
Girl	49 (52)	13 (42)	17 (52)	14 (50)	0.93 (0.54, 1.60)	0.799
Babies birth wgt (g) (mean)(sd)	3405 (445)	3843 (442)	3599 (499)	3600 (666)	1.07 (1.01, 1.13)	0.016^a^
Mode of delivery						
Normal	67 (71)	22 (71)	26 (79)	22 (79)		
Assisted	4 (4)	2 (6)	1 (3)	2 (7)	1.17 (0.34, 4.06)	0.810
Caesarean Section	24 (25)	7 (23)	6 (18)	4 (14)	0.65 (0.33, 1.27)	0.203
Randomised to wahakura						
No	48(51)	15(48)	18(55)	9(32)		
Yes	47(49)	16 (52)	15 (45)	19 (68)	1.33 (0.78, 2.28)	0.298

Proportional odds models were used to analyse the data

^a^The odds ratio is based on a difference in birthweight of 100 g

^b^The proportional odds model fitted as a trend across categories for the number of children

Table 3 shows frequencies, or in some cases the means (sd), of baseline variables for the four ‘fully breastfeeding’ time periods. The majority (92%) indicated they planned to ‘exclusively breastfeed’ and gave a planned duration of breastfeeding. Half planned to breastfeed for 24 weeks (IQR, 24–48) and the length of time they planned to breastfeed was positively associated with the duration breastfeeding (OR = 1.02, 95% CI 1.00, 1.03). The median for both questions asking about ‘the recommended time for exclusive breastfeeding’ of 48 weeks (IQR, 24–52) and the ‘introduction of solids’ of 24 weeks (IQR, 20–24), indicated that although breastfeeding knowledge was reasonable it was not predictive of breastfeeding duration.

**Table 3:** Means or frequency for breastfeeding planning, antenatal breastfeeding support, sleep quantity/quality for four ‘fully breastfeeding’ groups

	Full breastfeeding stopped before 4 weeks *N* = 95	Full breastfeeding stopped at/after 4 weeks but before 13 weeks *N* = 31	Full breastfeeding stopped at/after 13 weeks but before 26 weeks *N* = 33	Full breastfeeding stopped at/after 26 weeks *N* = 28	OR (95% CI)	*p*
Days before birth of child Mean (sd))	47 (25)	47 (24)	50 (23)	55 (25)		0.391
Plan to feed baby	n (%)	n (%)	n (%)	n (%)		
No	14 (18)	0 (0)	1 (4)	0 (0)		
Yes	81 (82)	31 (100)	32 (96)	28 (100)	15 (2, 118)	0.009
Plan to breastfeed (weeks) Mean (sd)^a^	29 (22)	34 (20)	40 (27)	41 (24)	1.02 (1.00,1.03)	0.012
Breast fed as baby						
Yes	68 (72)	20 (65)	24 (73)	22 (79)		
No	17 (18)	6 (19)	4 (12)	3 (11)	1.41 (0.66, 3.00)	0.368
Unsure	10 (11)	5 (16)	5 (15)	3 (11)	1.13 (0.51, 2.51)	0.758
Partner support (antenatal)						
Neutral/no support	23 (27)	6 (21)	4 (13)	2 (8)		
Strongly supportive	63 (73)	23 (79)	27 (87)	24 (92)	2.42 (1.15, 5.11)	0.02
Mother support (antenatal)						
Neutral/no support	25 (26)	7 (22)	7 (21)	2 (7)		
Strongly supportive	70 (74)	24 (77)	26 (79)	26 (93)	1.87 (0.96, 3.66)	0.067
Sleep Quantity Mean (sd)	2.6 (0.78)	2.8 (0.73)	2.4 (0.87)	2.6 (0.80)	1.01 (0.72. 1.42)	0.939
Sleep Quality Mean (sd)	2.8 (0.66)	2.9 (0.72)	2.9 (0.61)	2.9 (0.68)	1.22 (0.81, 1.83)	0.349

Proportional odds models were used to analyse the data

^a^Based on 66 participants in first group, 29 in the second, 24 in the third and 23 in the fourth

Before the birth of the child, 92% of mothers planned to breastfeed and the odds ratio for the duration of breastfeeding was strong (OR = 15; 95% CI 2, 118) but imprecise. Having a strongly supportive partner at the baseline interview (OR = 2.42; 95% CI 1.15, 5.11) was predictive of extended duration of breastfeeding. While there was trend for a similar support of breastfeeding by the mother of the participants to be predictive, this was not statistically significant. ‘Being breastfed as a baby’ was not associated with breastfeeding patterns.

At one month, having a ‘strongly supportive partner’ (OR = 3.64; 95% CI 1.76, 7.55) and a ‘strongly supportive mother’ (OR = 2.37; 95% CI 1.27, 4.82) was associated with extended breastfeeding. ‘Fatigue’ and ‘having others care for the baby’ was not. Both maternal smoking and the use of alcohol were associated with shorter duration of breastfeeding and the proportional-odds for the trend across the smoking categories was significant (OR = 0.51; 95% CI 0.37, 0.69; *p* < 0.001) (Table 4).

**Table 4:** Means or frequency for breastfeeding support and smoking at 1 month for four ‘fully breastfeeding’ groups

	Full breastfeeding stopped before 4 weeks *N* = 95	Full breastfeeding stopped at or after 4 weeks but before 13 weeks *N* = 31	Full breastfeeding stopped at or after 13 weeks but before 26 weeks *N* = 33	Full breastfeeding stopped at or after 26 weeks *N* = 28	OR (95% CI)	*p*
Age (days) at 1 m interview (Mean) (sd)	54 (29)	52 (28)	48 (17)	46 (15)		0.101
Breastfeeding support	n (%)	n (%)	n (%)	n (%)		
Strong partner support						
Neutral/no support	31/89 (45)	6/31 (19)	4/33 (12)	2/27 (7)		
Strongly supportive	58/89 (65)	25/31 (81)	29/33 (88)	25/27 (93)	3.64 (1.76, 7.55)	0.001
Strong mother support						
Neutral/no support	30/90 (33)	6(31 (19)	9/33 (28)	1/27 (4)		
Strongly supportive	60/90 (67)	25/31 (81)	24/33 (72)	26/27 (96)	2.47 (1.27, 4.82)	0.008
Others helped mother care for baby last night						
No	80/91 (88)	25/31 (19)	27/33 (22)	22/27 (81)		
Yes	11/91 (12)	6/31 (19)	6/33 (18)	5/27 (19)	1.50 (0.73, 3.11)	0.272
Fatigue (mean)(sd)	7.2 (4.59)	6.1 (2.67)	7.2 (5.56)	6.1 (4.79)^a^	0.97 (0.91, 1.03)	0.324
Maternal smoking						
None	37 (41)	21 (68)	25 (76)	21 (78)		
Occasional	9 (10)	0 (0)	2 (6)	1 (4)		
Daily	45 (49)	10 (32)	6 (18)	5 (19)	0.51 (0.37, 0.69)^b^	< 0.001
Alcohol use						
No	31/90 (48)	18 (58)	20/33 (61)	14/27 (56)		
Yes	59/90 (62)	13/31 (42)	13/33 (39)	13/27 (44)	0.54 (0.31, 0.93)	0.026

Proportional odds models were used to analyse the data

^a^Based on 91 participants in first group, 31 in the second, 33 in the third and 27 in the fourth

^b^Fitted as a trend across the categories

Data collected at all three postnatal surveys divided into the four breastfeeding groups mentioned above is shown in Table 5. To demonstrate the frequency for sleep place, bed sharing, pacifier use and sleep quantity and quality we employed a statistical model which allowed for time dependent covariates. The fourth group (mothers fully breastfeeding at 26 weeks) was censored, that is, not included in the analysis. Analysis showed that use of a pacifier was strongly associated with shortened periods of breastfeeding (OR = 0.28 95% CI 0.17, 0.46). Sleep place, bedsharing, maternal sleep and ‘possible depression’ were not associated with breastfeeding duration.

**Table 5:** Frequency for sleep place, bed-sharing, dummy use, sleep quantity and quality and depression against duration of ‘full breastfeeding’

	Breast feeding from birth *N* = 182	Still Breast feeding from 4 weeks or more *N* = 90	Still Breast feeding 13 weeks or more *N* = 61	Still Breast feeding 26 weeks or more *N* = 28	OR (95% CI)	*p*
	n (%)	n (%)	n (%)	n (%)		
Shared parent bedroom^a^	161 (82)	70 (78)	44 (72)	21 (75)	0.75 (0.42, 1.33)	0.324
Shared mother’s bed^a^	16 (9)	13 (14)	8 (13)	3 (11)	0.64 (0.33, 1.23)	0.179
Used dummy^a^	68 (37)	19 (21)	7 (11)	1 (4)	0.28 (0.17, 0.46)	< 0.001
Sleep quantity and	2.5 (0.65)	2.7(0.61)	2.7 (0.65)	2.5 (0.69)	0.91 (0.64, 1.29)	0.609
sleep quality (4 pt. scale)^a^	3.2 (0.54)	3.2 (0.51)	3.2 (0.60)	3.3 (0.70)	1.14 (0.74, 1.75)	0.549
‘Possible depression’ ^b^ (10+ on the EPDS)	65 (33)	10 (11)	4 (15)	2 (7)	0.92 (0.54, 1.57)	0.758

Cumulative odds models were used to analyse the data

^a^using data collected at all three postnatal assessments

^b^using data collected at baseline and 3 and 6 months

## Discussion

In this study we showed that in a predominantly Māori population drawn largely from deprived communities, the key predictors for extended duration of breastfeeding were the strong support for breastfeeding of the woman’s partner and her mother at one month postnatal, an intended duration of breastfeeding and being an older mother. Interestingly, whilst antenatal breastfeeding support by the participant’s mother was not predictive, support by the partner was, and although antenatal planning to breastfeed was a strong predictor, it was almost universal and therefore too imprecise to be useful. The key predictors for shorter duration of breastfeeding were pacifier use, daily cigarette smoking and increasing number of cigarettes per day, alcohol use and living in a more deprived area. Māori women were more likely to breastfeed for a shorter duration than non-Māori women.

We found the breastfeeding was extended by the strong support (for breastfeeding) of the woman’s partner and her mother, a higher intended duration of breastfeeding and being an older mother. Other studies have also identified the importance of partner and mother support for the duration of breastfeeding [12, 32] and family, peer and partner support for a pregnant woman’s intention to breastfeed [7]. New Zealand qualitative studies of infant feeding decisions by young Māori women indicate that, while mothers perceived breastfeeding as natural, easy, normal and healthy, it was “early stage breastfeeding support” in particular that was crucial to the development and maintenance of breastfeeding [22, 24]. We also found that breastfeeding duration increased with maternal age; in this case, year by year from 25 years to 29 years, contrary to results of a study from the United States with deprived inner city teen mothers [33].

We note that our study participants reported a median duration of full breastfeeding of only eight weeks, with only 18.7% exclusively breastfeeding at 13 weeks. These figures are lower than those reported nationally in New Zealand and far lower than the recommended “exclusive breastfeeding until six months of age” [28]. We propose that the very early introduction of solids that we have described played a role in this poor outcome. Our finding that 5% of mothers had introduced solids by one month is also of concern given the growing body of evidence relating early introduction of solids to a range of later life health issues, notably obesity [34, 35].

We found Māori ethnicity to be a negative predictor of breastfeeding duration, but we think that this is unlikely to be a factor per se; rather, it is a part of the complex web linking ethnicity, deprivation and cigarette smoking, the latter being of high prevalence in the Māori community. This is one of New Zealand’s ‘hard-to-change’ public health issues particularly amongst pregnant Māori women [36]. Tobacco use is known to be more prevalent in more deprived communities [37] and deprivation is a known predictor of early discontinuation of breastfeeding, interacting strongly with other socio-demographic factors, including age, education and ethnicity [7]. Maternal smoking and socio-economic status were both negative predictors for breastfeeding duration in our study. We also confirmed the dose relationship shown by others [38], that heavier smokers breastfeed for shorter periods. Whilst there may be physiological explanations for the relationship between smoking and breastfeeding, there are also complex psycho-social reasons [13], not least of which is mothers’ perceived risk of harm to their baby through tobacco’s toxic and addictive substances in their breast milk [14]. In response, some women stop breastfeeding if smoking cessation is too difficult. This same behaviour has also been found amongst Māori women [24].

Our study suggests that alcohol use may reduce breastfeeding duration. Infants are known to have decreased milk intake after their mothers have consumed alcohol [39]. Additionally, it is suggested that women might believe it better to stop breastfeeding if they drink alcohol in order not to harm baby [13] in the fashion suggested above for smoking. We also found pacifier use to be a very strong negative predictor of breastfeeding duration. While a meta-analysis investigating the protective effect of pacifiers for sudden infant death syndrome, found that the later use of a pacifier (after 4 weeks of age) did not impact on long term breastfeeding rates [40], other studies have noted that early and frequent, but not occasional, pacifier use shortens breastfeeding duration [18, 19, 41]. Our findings therefore support New Zealand’s stance to not promote pacifier use for the prevention of sudden infant death [42] as breastfeeding is protective of sudden unexpected death of an infant [43].

Breastfeeding education [44], in particular breastfeeding education for fathers [45], has been shown to enhance breastfeeding duration. However, despite evidence of reasonable breastfeeding knowledge amongst our participants, we did not demonstrate an association between knowledge of breastfeeding and duration of breastfeeding. Similarly, while being breastfed as a baby has been found to extend breastfeeding [46], we found no such association in our study. Others have found bedsharing to be a predictor of increased duration of breastfeeding [47] but we did not, and nor did a study in an inner city low income community in the United States of America [48]. In our study however, there were only small numbers bedsharing, given that participants were provided a wahakura or bassinet and asked to use it, but it could also be that bedsharing in this more deprived demographic is motivated by other factors rather than facilitation of breastfeeding. In a similar fashion, we did not find in this analysis that being assigned a wahakura extended breastfeeding duration as we did in our previous study [49]. This may be due to the more complex breastfeeding history collected and analyzed for this paper. Lastly, we found no association of depression in pregnancy with duration of breastfeeding although a systematic review of the literature [50] noted the association of depression during pregnancy and in the postpartum period with a shorter duration of breastfeeding.

The main strength of this study despite the low response rate was that it succeeded in recruiting a large sample of predominantly Māori women from deprived communities who we know are often reluctant to participate in research [51] particularly given the invasive nature of the full study, with cameras in the bedroom. Secondly, retention in the study was high, with 88% of those recruited still participating at the six-month interview. Thirdly, the breastfeeding history collected at each assessment was quite detailed and thus meant that the extent and length of breastfeeding could be robustly investigated. The reliability of data collected by phone at six months remains high as the research nurse had a well-established relationship with the participant.

Its main weakness, the possibility of recall bias around the breastfeeding duration, is offset somewhat by this study having used more than one question to identify behaviors, such as the use of ‘food not breast milk’ and ‘the introduction of solids’, rather than a simple 24 h food recall. Although our sample was not entirely Māori and Māori breastfeeding duration was shown to be shorter than non-Māori, we are assuming the predictors of breastfeeding duration described here are still relevant for a Māori population. Lastly, although the data is now five years old, the findings are relevant as the duration of breastfeeding in New Zealand has remained static since that time [52].

## Conclusion

This study of mainly Māori women from a relatively deprived community in New Zealand identified a shorter breastfeeding duration than other New Zealand women and confirmed the known negative predictors of breastfeeding duration, maternal smoking, alcohol consumption, pacifier use, and the early consumption of solids. Strong maternal and partner support of the mother was the strongest positive predictor of extended breastfeeding duration. Antenatal and postnatal education that includes the mothers and the partners of pregnant women and focuses on the identified predictors could be an effective strategy to increase breastfeeding in this population.

## Abbreviations

CI: Confidence intervals
IQR: Interquartile range
OR: Proportional odds ratio
